# Dynamics of *Campylobacter* spp. Contamination in Broilers from Farm-to-Slaughter Chain

**DOI:** 10.3390/pathogens15080803

**Published:** 2026-07-30

**Authors:** Carla Miranda, Miguel Sousa, Ana Cardoso, Sérgio Afonso, Mariana Caipira Lei, Nuno V. Brito

**Affiliations:** 1 University Institute of Health Sciences (IUCS), Polytechnic and University Higher Education Cooperative (CESPU), Avenida Central de Gandra, 1317, 4585-116 Gandra, Portugal; miguel.sousa@cespu.pt (M.S.); ana.cardoso@cespu.pt (A.C.); a31380@alunos.cespu.pt (S.A.); mariana.lei@iucs.cespu.pt (M.C.L.); nuno.brito@iucs.cespu.pt (N.V.B.); 2 UCIBIO—Applied Molecular Biosciences Unit, University Institute of Health Sciences (1H-TOXRUN, IUCS-CESPU), Avenida Central de Gandra 1317, 4585-116 Paredes, Portugal; 3 Associate Laboratory i4HB—Institute for Health and Bioeconomy, University Institute of Health Sciences CESPU, Rua Central de Gandra, 1317, 4585-116 Gandra, Portugal; 4 LAQV-REQUIMTE—Associated Laboratory for Green Chemistry, University NOVA of Lisbon, 1099-085 Caparica, Portugal; 5 Center for the Research and Technology of Agro-Environmental and Biological Sciences (CITAB), University of Trás-os-Montes e Alto Douro (UTAD), 5000-801 Vila Real, Portugal; 6 CISAS—Center for Research and Development in Agrifood Systems and Sustainability, Polytechnic Institute of Viana do Castelo, Rua Escola Industrial e Comercial de Nun’Álvares, 4900-347 Viana do Castelo, Portugal

**Keywords:** *Campylobacter*, broiler, biosecurity, antimicrobial resistance, food safety, One Health

## Abstract

*Campylobacter jejuni* and *C. coli* are leading causes of bacterial gastroenteritis worldwide, commonly linked to undercooked poultry. Within a One Health perspective, understanding their dissemination from farm-to-slaughterhouse is essential. This study aimed to detect *Campylobacter* spp. in intensively reared broiler chickens at critical points along the production chain and to assess antimicrobial resistance genes. Two production cycles, November to December 2024 and July to August 2025, were monitored weekly across three Portuguese poultry farms and a shared slaughterhouse. In total, 2164 samples were collected, including farm, transport, and slaughterhouse samples. Detection followed ISO 10272-1:2017 and 10272-2:2017 standards. *Campylobacter* prevalence peaked during weeks 3 and 4 on farms (9.4% cloacal, 23.5% litter). Transport crates showed contamination before/after loading (15.0%). At the slaughterhouse, contamination was low (8.3%) in process waters but high in neck skin samples (76.7%), with 15.2% exceeding the 1000 CFU/g limit (EU Regulation 2017/1495). Among 56 confirmed isolates, *C. jejuni* was most prevalent (46.4%), followed by *C. coli* (32.1%). The *tet*O gene was detected in most isolates (69.4%), while *bla*_OXA-61_ was less frequent (30.6%). Findings highlight the need for integrated control strategies, including improved biosecurity, crate sanitation, optimized processing procedures, systematic monitoring, and responsible antimicrobial use to enhance food safety and public health.

## 1. Introduction

*Campylobacter* spp. infection is a widespread infectious disease that significantly impacts global public health, often leading to gastroenteritis and, in some cases, severe autoimmune conditions. The incidence and prevalence of campylobacteriosis have risen over the past decade in both developed and developing countries, with particularly alarming increases observed in North America, Europe, and Australia [1,2]. In the European Union, campylobacteriosis has been the most reported foodborne zoonosis since 2005, with 148,181 confirmed human cases in 2023 alone, representing 58.9% of all notified zoonotic infections [3]. Most human infections, approximately 80 to 90%, are attributed to *C. jejuni*, while *C. coli* accounts for the remaining 10 to 20% of cases [4]. Beyond the acute diarrheal illness, *Campylobacter* infection may lead to severe post-infectious sequelae, including Guillain-Barré syndrome (0.1% of cases), inflammatory bowel disease (0.4%), reactive arthritis (1.7%), and irritable bowel syndrome (4.5%), thereby underscoring its considerable clinical and economic significance [5].

Transmission occurs predominantly through the foodborne route, with the consumption of undercooked poultry meat identified as the principal risk factor in epidemiological investigations. The European Food Safety Authority (EFSA) estimates that chicken and chicken products account for approximately 20–30% of human campylobacteriosis cases [2]. The industrial poultry production chain constitutes the primary epidemiological reservoir of *Campylobacter* [6,7]. In broiler flocks, *C. jejuni* and *C. coli* establish themselves as asymptomatic commensals, predominantly colonizing the cecum and lower gastrointestinal tract without causing apparent clinical disease. Colonization typically begins between 2 and 3 weeks of age, reaching prevalence of 50–90% in flocks at slaughter. Rapid intra-flock dissemination occurs via horizontal transmission, including coprophagy, bird-to-bird contact, and environmental contamination, resulting in widespread contamination of carcasses, neck skins, and by-products at the slaughterhouse [6,8,9].

The associated public health risk in primary production can be mitigated through biosecurity measures, including strict access control, potable water supply, thorough cleaning between flocks, and all-in/all-out slaughter management, which have demonstrated significant efficacy in reducing *Campylobacter* flock colonization [8,10]. Complementing these interventions at farm level, Commission Regulation (EU) 2017/1495 established a process hygiene criterion for *Campylobacter* spp. on broiler carcasses, setting a critical limit of 1000 CFU/g on neck skin. The regulation stipulates a sampling plan of 50 samples per collection period, with the maximum number of non-compliant results progressively reduced from c = 20 in 2018 to c = 15 in 2020, and to c = 10 from January 2025 onwards [11]. Risk assessments conducted by the EFSA have estimated that full compliance with this criterion could reduce the incidence of human campylobacteriosis by approximately 50% [2].

In addition to efforts aimed at reducing the presence of *Campylobacter* spp. in poultry meat and related by-products, this commensal bacterium can harbor antimicrobial resistance genes. Concurrently, antimicrobial resistance in *Campylobacter* spp. represents an escalating concern for both public and animal health [12]. Tetracyclines remain among the most sold antimicrobial classes for food-producing animals in the EU, accounting for 21.6% of total sales in 2023 [13], and their continued use in poultry production contributes substantially to the selection and spread of resistant strains, with direct implications for therapeutic options in human medicine. Resistance to tetracyclines, mediated by the ribosomal protection protein gene *tet*O, and resistance to β-lactams, conferred by the class D β-lactamase gene *bla*_OXA-61_, have been documented in isolates of poultry origin. Prevalence rates of 52% for *tet*O and 72% for *bla*_OXA-61_ have been reported in *Campylobacter* spp. isolated from broilers and humans [14]. Fluoroquinolones (e.g., enrofloxacin) and macrolides (e.g., erythromycin) are considered the antimicrobial classes of greatest clinical relevance for campylobacteriosis, as they represent the first-line therapeutic options for treating this infection in humans; consequently, resistance to these classes is of particular concern when documented in *Campylobacter* isolated from broiler chickens [12,13]. In recent studies, *C. jejuni* isolated from chickens and slaughterhouse workers in Algeria showed 82.6% resistance to ampicillin, 49.6% to nalidixic acid, and 33% to erythromycin, while *C. coli* exhibited 50% resistance to ciprofloxacin, 38.9% to tetracycline, and 40.7% to nalidixic acid [15]. Consistently, in chicken meat samples of Baltic origin, 90.2% of *Campylobacter* strains were resistant to nalidixic acid and ciprofloxacin, 57.4% to tetracycline, 42.6% to streptomycin, and 6.6% to erythromycin, while all remained sensitive to gentamicin [16]. Consequently, the integrated assessment of *Campylobacter* contamination along the farm-to-fork continuum, together with the characterization of antimicrobial resistance profiles, is an essential component of a One Health approach to food safety.

Despite extensive documentation on *Campylobacter* prevalence in broiler chickens and its public health impact, studies featuring representative sampling (n > 1000) that longitudinally monitor contamination from farm to slaughter remain scarce. This limitation hinders the integrated characterization of species-specific prevalence (*C. jejuni* and *C. coli*), bacterial load dynamics, and antimicrobial resistance profiles throughout the “farm-to-fork” production chain. To address this gap through intensive monitoring of poultry farms and their associated slaughterhouse, this study aimed to detect and epidemiologically characterize *Campylobacter* spp., with an emphasis on *C. jejuni* and *C. coli*, and to identify the resistance genes *tet*O and *bla*_OXA-61_, thereby identifying critical intervention points to enhance biosecurity measures and antimicrobial stewardship policies.

## 2. Materials and Methods

### 2.1. Study Design and Locations

**Figure 1:**
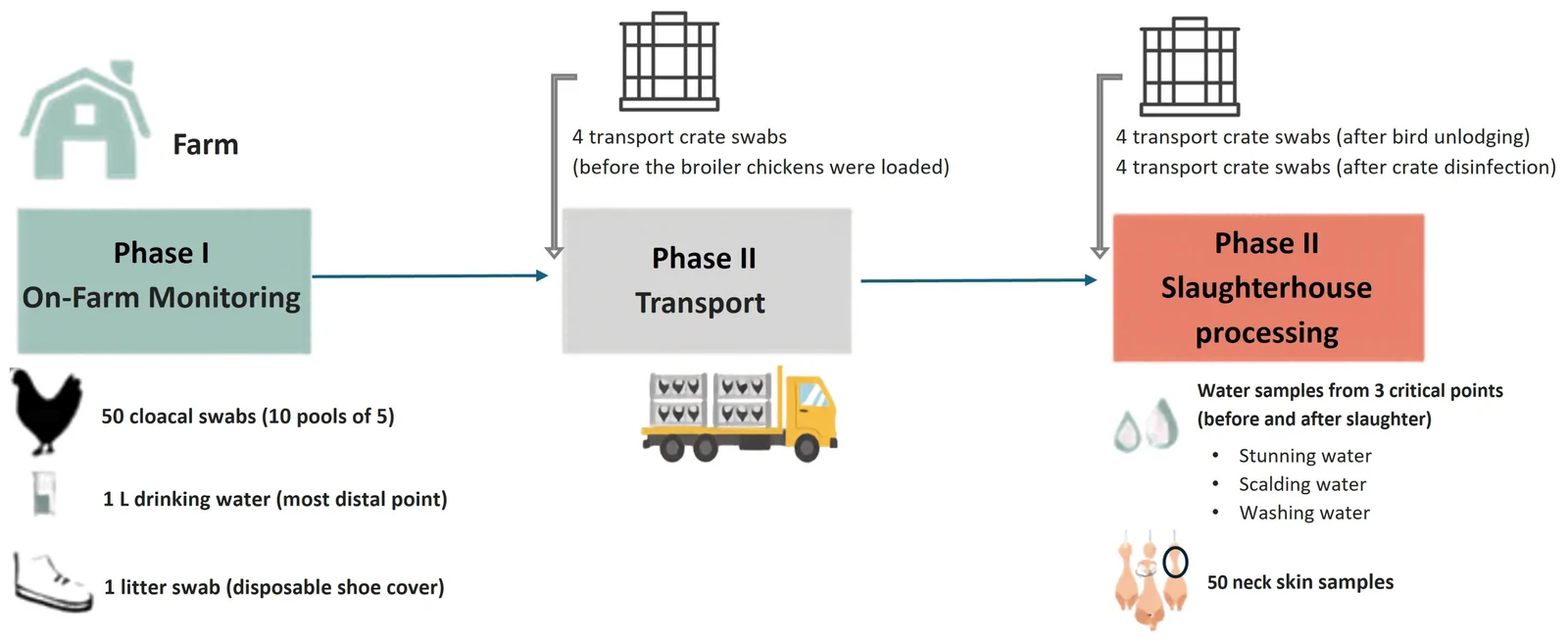
Experimental design showing the monitored phases of the study: on-farm, transport and slaughterhouse, as well as sample collection.

An observational study was conducted on three intensive broiler chicken farms, Farms A, B, and C. These were selected based on geographical proximity. One flock per farm was randomly selected, along with the destination slaughterhouse located in Portugal. Monitoring took place over two production cycles: November–December 2024 (cycle 1) and July–August 2025 (cycle 2), reflecting distinct seasonal conditions frequently associated with variations in campylobacteriosis incidence [17]. Each cycle encompassed the monitoring of three distinct phases: on-farm, transport, and slaughterhouse. Flocks were monitored from day 1 of age until slaughter, with weekly sampling at six time points (T0: day 1; T1: day 8; T2: day 15; T3: day 22; T4: day 29; T5: day 36), followed by additional collections during transport and at the slaughterhouse (Figure 1).

Each flock was housed under intensive, floor-based management using Ross 308 broilers. Initial flock sizes varied across farms and cycles: Farm A, Farm B, and Farm C housed 41,000, 23,460, and 70,000 broilers, respectively, in cycle 1; while in cycle 2, the flocks were 44,000, 39,500, and 39,000 broilers, respectively. Throughout the rearing period, progressive reductions in flock size were observed across all farms, attributable to cumulative mortality (approximately 2.5–8.0%). Flock health and sanitary data, including daily mortality rates, clinical history, and veterinary treatments, were systematically monitored and retrieved from the official farm records and herd registers. All flocks were reared under standard intensive commercial conditions under the supervision of the respective farm veterinarians.

### 2.2. Farm Sampling

On-farm standardized weekly samples were collected from T0 to T5. At each sampling time point, 50 cloacal samples were randomly collected from 50 individual animals, and 1 protective disposable shoe-cover swab used by the technician during sampling was collected as a representative sample of flock litter. Since these 50 animals were selected at random from the total flock population at each sampling time, they represented a different cohort of individuals every week, establishing a repeated cross-sectional sampling design rather than a longitudinal follow-up of the same individual birds. The shoe cover was worn by the technician throughout the visit, including during water and cloacal sample collection, and a zig-zag walking pattern covering the entire house was performed at the end of each visit to ensure representative contact with the litter across the whole floor area. Both samples, cloacal and disposable shoe cover swab samples, were collected using sterile transport swabs with Amies charcoal medium (Cliniswab, Canelli, Italy). In addition, a 1 L drinking water sample was collected directly from the most distal point of the distribution system. Individual cloacal samples were pooled in groups of five, generating 10 pools per sampling time, while litter material and water samples were analyzed individually for the presence of *Campylobacter*. Flock densities were compatible with European intensive systems described in previous *Campylobacter* colonization studies [18].

### 2.3. Transport Sampling

*Campylobacter* dissemination was evaluated during the broiler transport between the farm and slaughterhouse. The crates used for loading chickens were sampled at three distinct time points: before broiler entry on-farm (on the same day as T5); immediately after transport, once the animals were unloaded at the slaughterhouse; and, in the second production cycle of all farms, after crate washing and disinfecting procedure.

At each flock and production cycle, 4 crate samples were collected at each time point, resulting in a total of 24 samples before transport, 24 after transport, and 12 after washing/disinfecting across the two study cycles. Samples consisted of swabs taken from the internal surfaces of crates in areas that directly contact broilers. Before transport, crates were randomly sampled and subsequently followed through to third sampling point. Samples consisted of swabs taken from the internal surfaces of crates in areas of direct contact with broilers, using the same sterile transport swabs with Amies charcoal medium (Cliniswab, Canelli, Italy) as those described for cloacal and shoe cover sampling. Each surface was manually swabbed by rubbing the swab back and forth 10–15 times to standardize sample recovery. All operators involved in crate sampling were trained by the same technician, who also handed over the swabs and explained the standardized procedure before sample collection.

### 2.4. Slaughterhouse Sampling

At the slaughterhouse, environmental and carcass contamination were evaluated by collecting water samples and neck skins. At each flock and cycle, 1 L of water samples were collected from three process steps, such as stunning tank, scalding tank, and final carcass washing system. At each step, samples were collected at two timepoints: before the start of slaughter of the study flocks and after their passage, totaling 36 water samples throughout the study. After chilling, approximately 25 g of neck skins were collected from 50 randomly selected carcasses per flock and cycle, pooled in groups of five (10 pools per flock by cycle).

### 2.5. Microbiological Processing and Identification of Campylobacter Sample

Analysis was performed according to standard methods for detecting *Campylobacter* spp., including ISO 10272-1:2017 (qualitative detection), ISO 10272-2:2017 (quantitative enumeration) [19,20], and the membrane filtration method (water) [21].

Briefly, pooled cloacal swabs, disposable shoe cover swabs (representative of litter), and crate swabs were pre-enriched in Bolton broth (BB; Oxoid, Basingstoke, UK) supplemented with 5% lysed horse blood (SR0048C) and selective supplement (SR0183) and incubated at 42 °C for 48 ± 4 h under microaerophilic conditions (5% O_2_, 10% CO_2_, 85% N^2^). Following the incubation period, 20 μL (corresponding to two loops) of each BB culture was streaked onto CHROMagar™ *Campylobacter* (CHROMagar, Paris, France) and modified Charcoal Cefoperazone Deoxycholate Agar (mCCDA supplemented with CM0739B and SR0155). Plates were incubated for 48 h at 42 °C under microaerophilic conditions, and presence/absence of typical colonies was recorded. Reference strains *C. jejuni* ATCC 33560 and *C. coli* ATCC 33559 served as controls.

Water samples, drinking water from farms and slaughterhouse water (stunning tank, scalding tank, carcass washing) were processed using the membrane filtration method: 100 mL of each sample was filtered through 0.45 μm cellulose nitrate membranes (Sartorius, Göttingen, Germany), and the membrane was then introduced into Bolton broth and processed qualitatively as described [21]. The pooled neck skin samples were stomached in buffered peptone water (BPW), using a Stomacher^®^ (Stomacher^®^ 400 Circulator, Seward Laboratory Systems Inc., Islandia, NY, USA), followed by decimal dilutions (10^−1^ and 10^−2^). Each dilution (0.1 mL) was plated onto mCCDA and incubated at 42 °C for 48 h under microaerophilic conditions. *Campylobacter* counts were expressed as CFU/g.

Presumptive *Campylobacter* colonies were subcultured and subjected to biochemical tests (Gram staining, oxidase, catalase). These isolates were cryopreserved in brain heart infusion broth supplemented with 5% lysed horse blood and 15% glycerol, at −80 °C for further analysis.

Bacterial DNA was extracted at 100 °C for 10 min, using the boiling method [22]. *Campylobacter* identification was confirmed by PCR assays, using the Xpert Fast Hotstart Mastermix (GRiSP Research Solutions, Porto, Portugal) according to the manufacturer’s instructions. Specific primer sequences targeting the 16S rRNA gene for *Campylobacter* spp. (CH412F/C1228R), the *ceu*E gene for *C. coli* (CC18F/CC519R), and the *map*A gene for *C. jejuni* (C1/C3), along with their respective annealing temperatures (Table 1), were based on previously published studies [23,24,25]. DNA was extracted from the reference strains *C. jejuni* ATCC 33560 and *C. coli* ATCC 33559, which served as control strains. The integrity of the extracted DNA was verified by agarose gel electrophoresis prior to downstream analyses, ensuring its suitability for amplification.

### 2.6. Detection of Antimicrobial Resistance Genes

Detection of antimicrobial resistance genes in *Campylobacter* isolates was performed by PCR, using the Xpert Fast Hotstart Mastermix (GRiSP Research Solutions, Porto, Portugal) according to the manufacturer’s instructions. Tetracycline resistance was assessed by targeting the *tet*O gene and *bla*_OXA-61_, which encodes a class D β-lactamase implicated in β-lactam resistance [13]. Primer sequences were based on previously published studies [26,27,28] (Table 2). Positive and negative controls were used.

### 2.7. Statistical Analysis

Descriptive statistics were used to summarize the microbiological counts of *Campylobacter* (CFU/g) across different farms and production cycles. Data were expressed as mean values ± standard deviation (SD) to serve as indicators of variance and to illustrate the magnitude and heterogeneity of carcass and environmental contamination. Due to the nature of the sampling and the expected non-normal distribution of microbial counts, the minimum and maximum ranges were also reported alongside the mean values to provide a comprehensive representation of data dispersion without assuming normality. Pearson’s correlation coefficient was used to assess the temporal association between *Campylobacter* positivity rates in pooled cloacal samples and litter material across the six sampling time points. The data were registered and analyzed using the MS Excel 2016 software. The collected data was registered on a table on an Excel sheet to create the different graphs that are presented, as well as to calculate all the percentages.

## 3. Results

### 3.1. Sampling Characterization

A total of 2164 samples were collected from six farm-to-slaughter chains, comprising two production cycles per farm. Among the three farms included in this study, 1768 samples were collected, including cloacal and disposable shoe-cover swabs and drinking water. Additionally, 60 samples were collected from crates during the broiler transport, and 336 samples were collected at the slaughterhouse, including 36 water samples from three distinct processing steps, and 300 neck skin samples (Table 3). In production cycle 1, 1024 samples were collected, a lower number than in production cycle 2, in which 1140 samples were collected. This variation in the sample size between cycles was due to two main factors. First, during production cycle 1 on farms B and C, on-farm monitoring ended earlier, with broilers being slaughtered immediately after sampling time point T4. Second, a methodological adjustment was implemented in production cycle 2, during which crate swabs collected after the washing and disinfection process were introduced across all farms.

### 3.2. Campylobacter in Farm Samples

**Figure 2:**
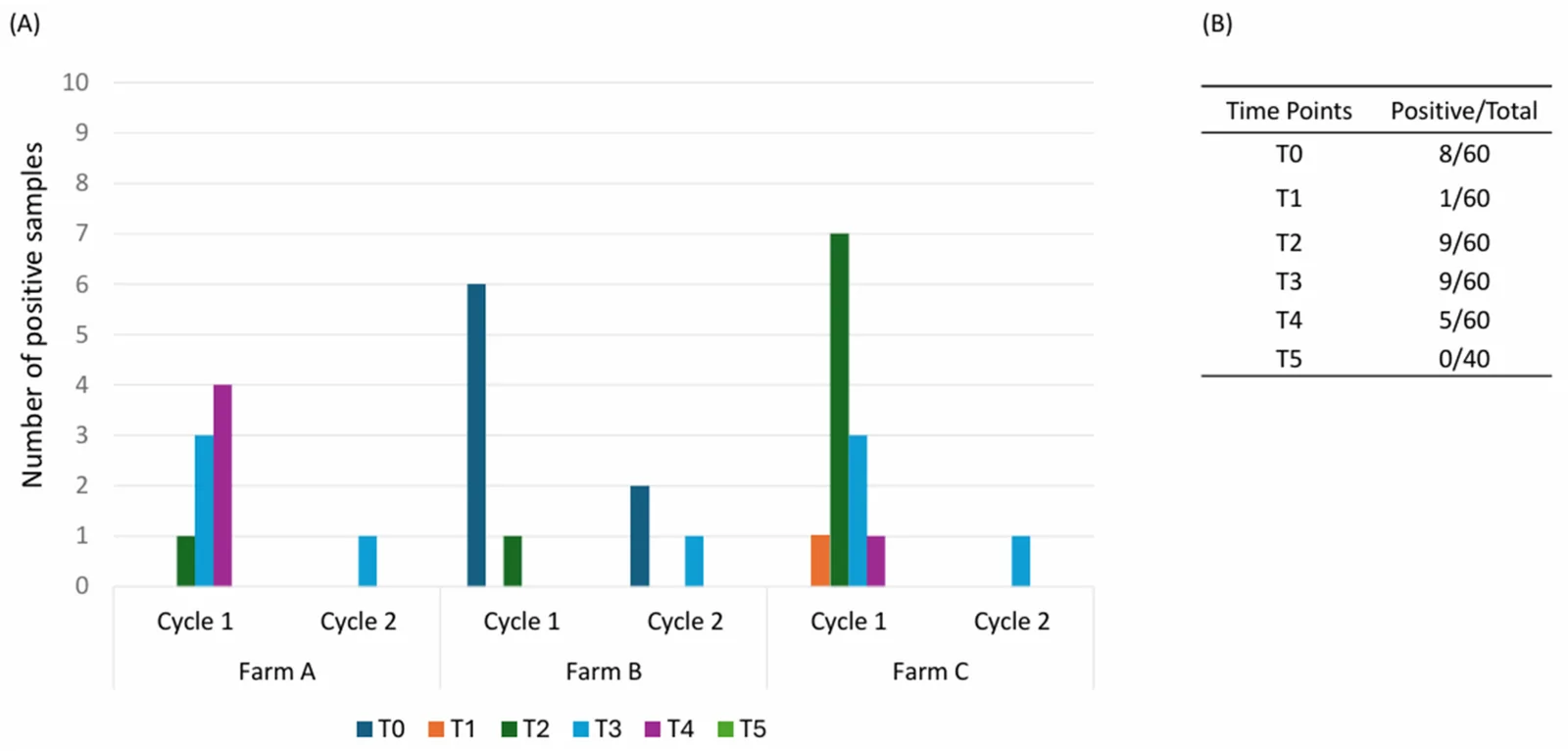
Number of positive-*Campylobacter* pooled cloacal samples across both production cycles (10 pools per cycle) for each farm. (**A**) Distribution of positive samples by sampling time (T0–T5) during cycle 1 (November–December 2024) and cycle 2 (July–August 2025). (**B**) Total ratio of positive samples relative to the total samples analyzed at each time point (Time Points T0–T5).

*Campylobacter* spp. presence on farms was assessed using pooled cloacal swabs, litter material collected with disposable shoe-cover swabs and drinking water samples across six weekly sampling time points (T0–T5). Overall, 9.4% (32/340) of pooled cloacal samples and 23.5% (8/34) of litter material samples were positive for *Campylobacter*, (Figure 2), whereas all (n = 34) drinking water samples were negative.

Among pooled cloacal samples, *Campylobacter* detection rates increased most markedly by day 15 of age (T2), with this rise observed in farms A and B, during both production cycles (Figure 2). When analyzed by sampling time, 2.4% (8/60) of cloacal samples were positive at T0, 0.3% (1/60) at T1, 2.6% (9/60) at both T2 and T3, and 1.5% (5/60) at T4, while all pooled cloacal samples collected at T5 were negative.

**Figure 3:**
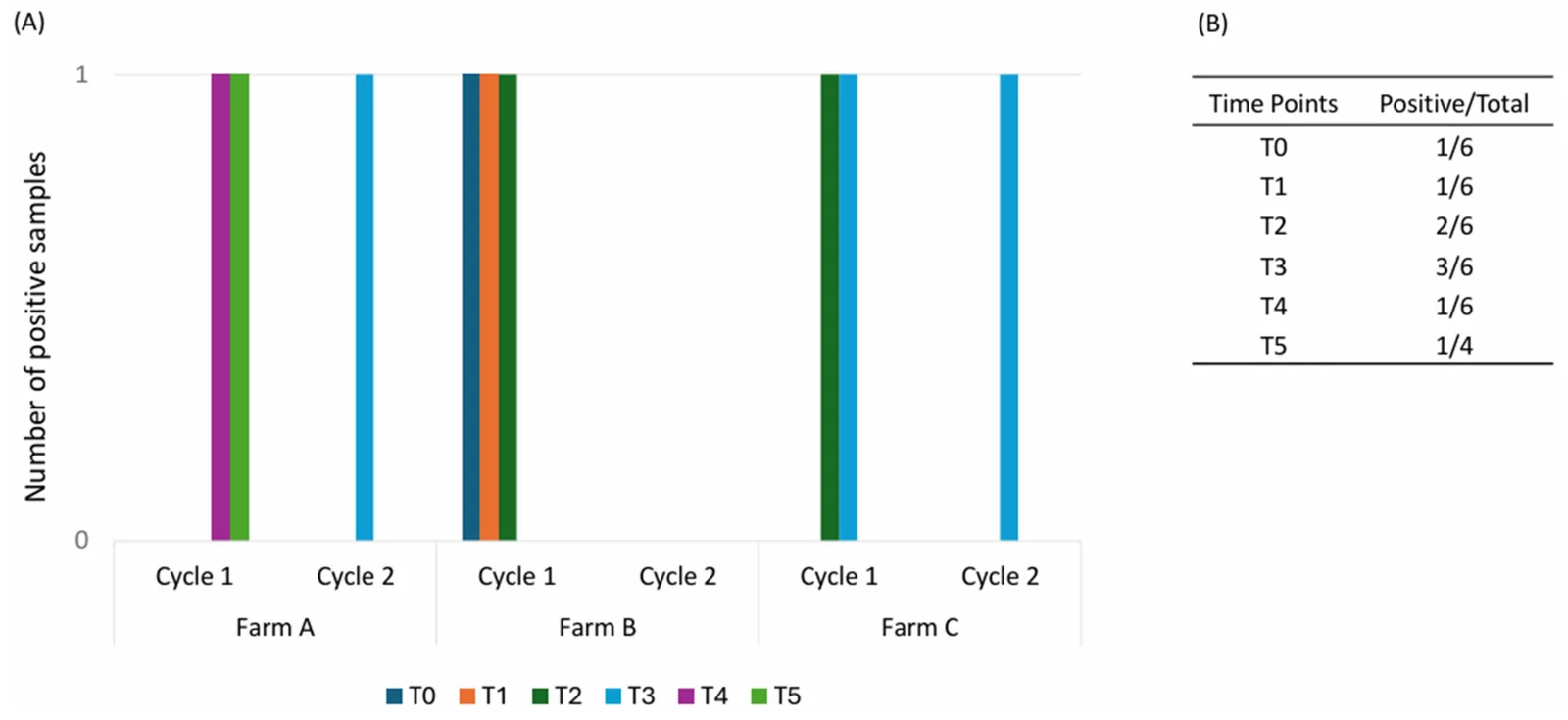
Number of positive-*Campylobacter* litter material samples (disposable shoe-cover swabs) across both production cycles (1 sample per cycle) for each farm. (**A**) Distribution of positive boot swab samples by sampling time (T0–T5) during cycle 1 (November–December 2024) and cycle 2 (July–August 2025). (**B**) Total ratio of positive samples relative to the total samples analyzed at each time point (Time Points T0–T5).

As observed for cloacal samples, *Campylobacter* was detected in litter material, with the highest number of positive samples at T3 (Figure 3). When analyzed by sampling time, 8.8% (3/6) of disposable shoe cover swab samples were positive at T3, whereas 2.9% (1/6) were positive at each of the remaining sampling times, T0–T2 and T4–T5. A positive Pearson correlation was observed between cloacal and litter positivity rates across the six sampling time points (r = 0.652).

### 3.3. Campylobacter in Crate Transport Samples

**Figure 4:**
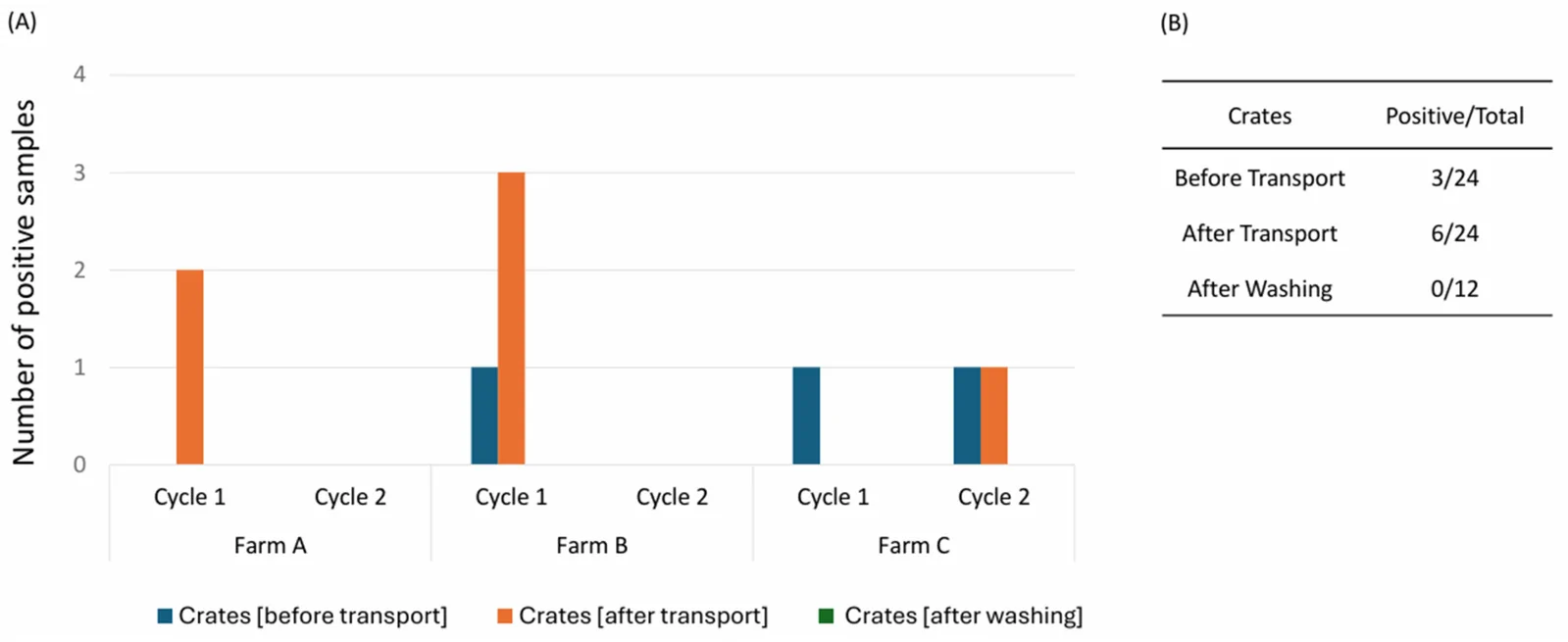
Number of *Campylobacter* spp. positive crate samples across both production cycles (4 crates per phase per cycle) for each farm. (**A**) Distribution of positive crate samples collected before and after broiler transport, and after washing of crates in the slaughterhouse during cycle 1 (November–December 2024) and cycle 2 (July–August 2025); (**B**) total ratio of positive samples relative to the total samples analyzed for each phase (Before Transport, After Transport, and After Washing).

The assessment of *Campylobacter* presence at three distinct stages of broiler transport showed that 15.0% (9/60) of crate swab samples were positive. Of these, 5.0% (3/24) were positive before animal transport, and 10.0% (6/24) after animal transport (Figure 4).

In production cycle 1, contaminated crates were detected both before and after transport across all three farms (Figure 4). In farm C, results were consistent across both production cycles, with *Campylobacter* detected in crate swabs collected before animal transport. In production cycle 2, the inclusion of sampling immediately after the washing/disinfection process performed at the slaughterhouse demonstrated absence of *Campylobacter*.

### 3.4. Campylobacter in Slaughterhouse Samples

**Figure 5:**
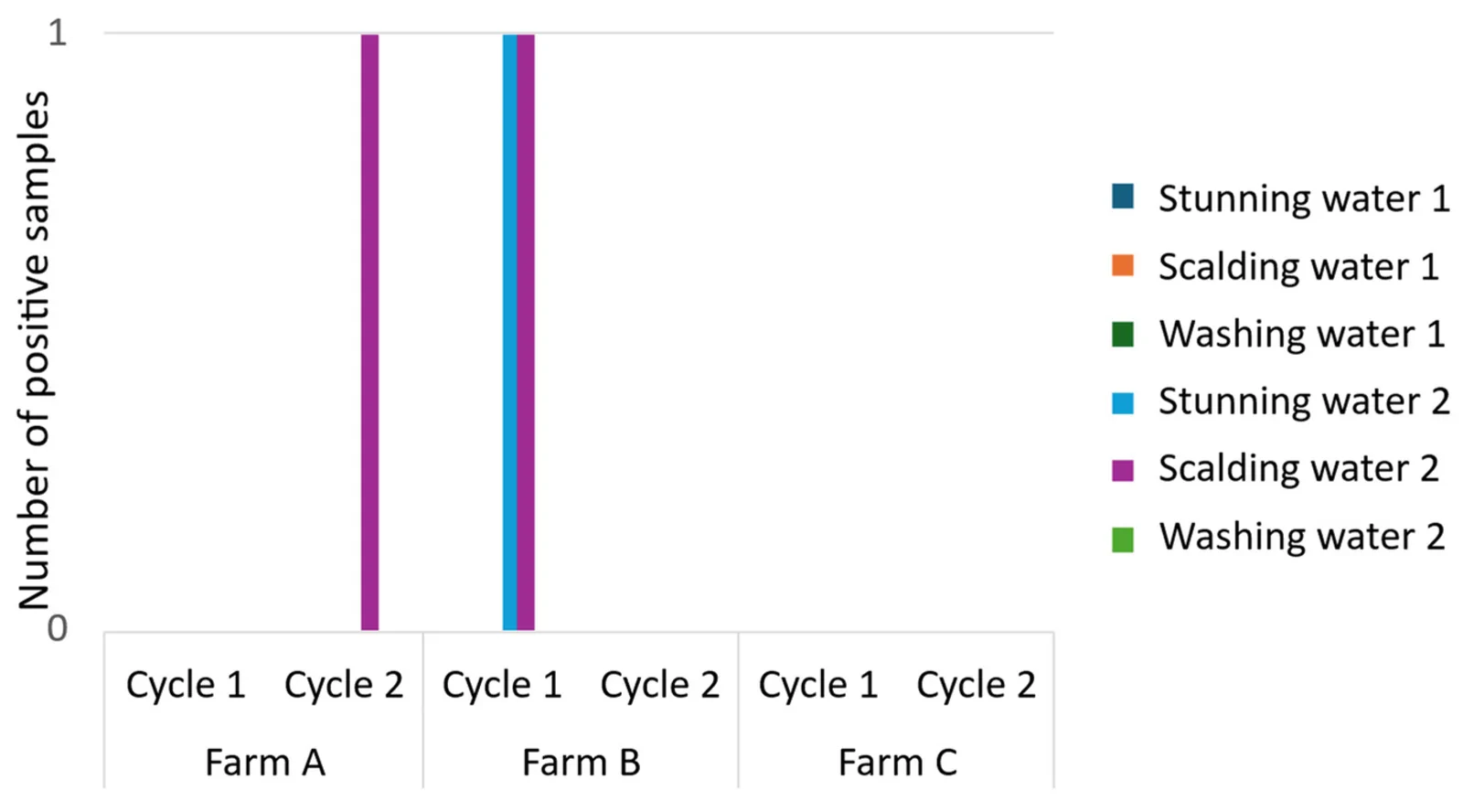
Number of water samples positive for *Campylobacter* collected during the slaughter of the study flocks, by production cycle 1 (November–December 2024) and cycle 2 (July–August 2025), for each farm. Stunning water 1, scalding water 1, and washing water 1 samples were collected before the passage of the broiler flocks. Stunning water 2, scalding water 2, and washing water 2 samples were collected after their passage through the respective slaughterhouse steps.

At the slaughterhouse, 36 water samples were collected from the stunning, scalding, and carcass washing points, before and after slaughter of the study flocks (Figure 5). Overall, 8.3% (3/36) of the samples were positive for *Campylobacter*, all collected after slaughter, specifically from stunning water and scalding waters.

Sporadic *Campylobacter* detection was observed after flock passage, occurring primarily in scalding water in production cycle 1 from farm flock B and in production cycle 2 from farm flock A (Figure 5).

Broiler carcass contamination was assessed by quantifying *Campylobacter* in pooled neck skin samples collected from each flock cycle per farm, with reference to the 1000 CFU/g threshold defined in the Regulation (EU) 2017/1495. Overall, 76.7% (46/60) of the pooled neck skins were positive for *Campylobacter*, although 84.8% of the counts remained below 1000 CFU/g, classifying them as satisfactory, according to this regulation (Table 4).

The presumptive counts of *Campylobacter* from pooled neck skin samples ranged from 0 to 2100 CFU/g for Farm A, 0 to 300 CFU/g for Farm B, and 0 to 2600 CFU/g for Farm C, demonstrating heterogeneity in carcass contamination (Table 4). Both Farms A and C showed an increase in the number of presumptive positive samples and mean microbial loads from production cycle 1 to cycle 2; however, this increase was most pronounced in Farm C, where the mean load rose from 130 ± 149 CFU/g to 1090 ± 856 CFU/g. During cycle 2 (July–August), all samples from Farms A and C yielded presumptive colonies, with multiple pools exceeding 1000 CFU/g. Conversely, Farm B showed a decrease in the number of positive pools (from 9 to 4) and a reduction in the mean microbial level from 170 ± 95 CFU/g in cycle 1 to 80 ± 114 CFU/g in cycle 2. Subsequent molecular analysis via PCR was performed to confirm the definitive identity of these isolates.

### 3.5. Campylobacter Identification

**Figure 6:**
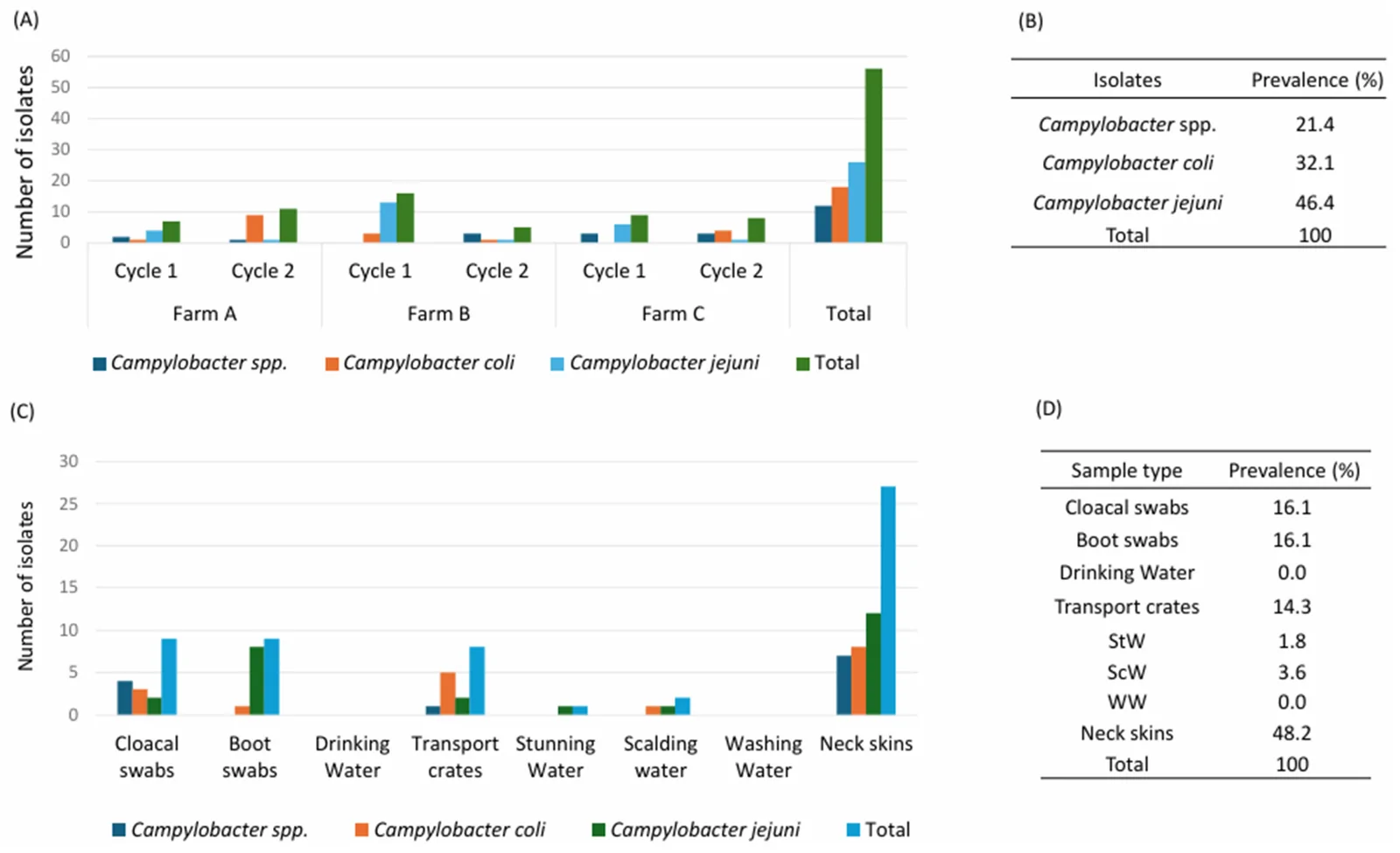
Distribution of *Campylobacter* isolates identified in this study (n = 56). (**A**) distribution by farm and production cycle, including (**A**) number of isolates by *Campylobacter* species across production cycle 1 and cycle 2 for each farm and (**B**) prevalence percentage of isolates by species. (**B**): Distribution by matrix, showing: (**C**) number of isolates across different sample types, including cloacal swabs, boot swabs, drinking water, transport crates, slaughterhouse water as stunning (StW), scalding (ScW), and washing (WW), and neck skin samples and (**D**) prevalence percentage of isolates obtained for each sample type.

A total of 144 presumptive isolates displaying typical *Campylobacter* spp. morphology were collected across all sampling matrices (farm, crates, litter, slaughterhouse, and neck skin). Of these, 56 *Campylobacter* isolates selected based on farm and production cycle, were identified using PCR assay, with 18 isolates obtained from farm A, 21 from farm B and the remaining 17 from farm C. The following species were identified among these isolates: 46.4% (n = 26) were *C. jejuni*, 32.1% (n = 18) were *C. coli*, and 21.4% (n = 12) were classified as *Campylobacter* spp. (Figure 6), supporting the results obtained by both culture methods.

In production cycle 1, the isolates were predominantly identified as *C. jejuni* across all farms, whereas *C. coli* isolates were more prevalent in production cycle 2, particularly in samples from farms A and C (Figure 6A). Overall, based on the type of samples collected from farm to slaughter, nearly half (48.2%) of *Campylobacter* isolates were obtained from neck skin samples, followed by cloacal and litter material (16.1% each), crates (14.3%), and scalding/stunning water (5.4%, collection after slaughter). *Campylobacter jejuni* was detected in cloacal, litter material and slaughterhouse samples, whereas *C. coli* was detected mainly in crate transport samples from farms B and C, and in neck skin samples from farm A (Figure 6B).

### 3.6. Antibiotic Resistance Genes

**Figure 7:**
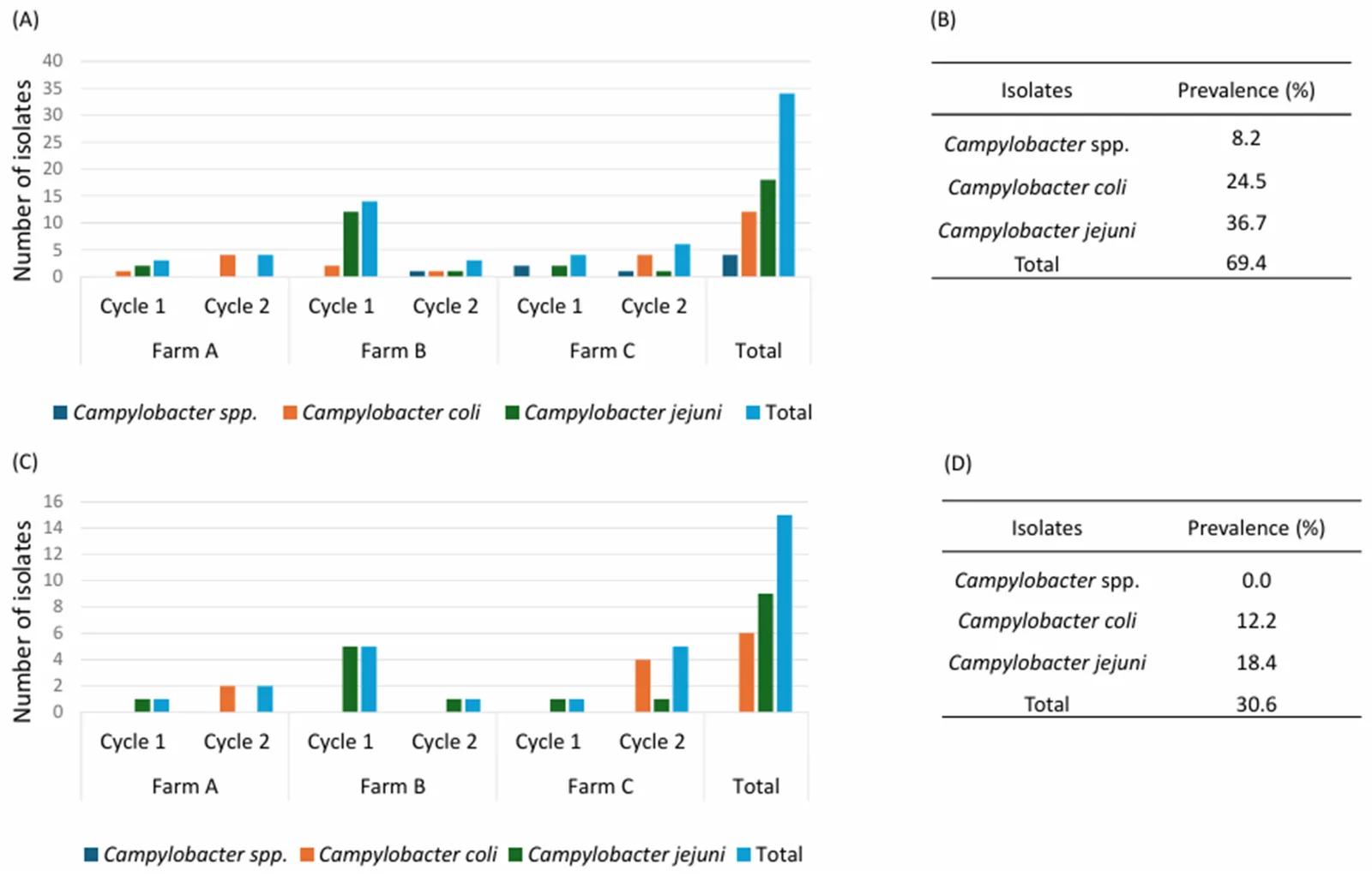
Antibiotic resistance genes identified in *Campylobacter* isolates in this study (n = 49). Distribution of the *tet*O gene, showing: (**A**) number of isolates by *Campylobacter* species, production cycle, and farm and (**B**) prevalence percentage of isolates carrying the *tet*O gene relative to the total population. Distribution of the *bla*_OXA-61_ gene, showing: (**C**) number of isolates by *Campylobacter* species, production cycle, and farm and (**D**) prevalence percentage of isolates carrying the *bla*_OXA-61_ gene relative to the total population.

Of the 56 *Campylobacter* isolates, 49 were subjected to PCR analysis to detect resistance genes, specifically *tet*O and *bla*_OXA-61_. The *tet*O gene, conferring resistance to the tetracycline class of antimicrobials, was detected in 69.4% (34/49) of the isolates across all farms. Distribution by species showed that 36.7% (18/49) were identified as *C. jejuni*, 24.5% (12/49) as *C. coli* and 8.2% (4/49) as *Campylobacter* spp. In addition, doxycycline administration for omphalitis treatment was recorded during production cycle 1 at farm B, coinciding with the detection of *tet*O-positive isolates at that farm and in that production cycle (Figure 7A).

The *bla*_OXA-61_ gene, conferring resistance to the β-lactam antimicrobial class, was detected in 30.6% (15/49) of the isolates, across all farms. Based on species identification, 18.4% (9/49) of the isolates were *C. jejuni*, and 12.2% (6/49) were *C. coli*. No isolates were found to harbor both antibiotic genes simultaneously (Figure 7B).

## 4. Discussion

This study generated an extensive dataset comprising 2164 samples collected across multiple stages, including farm, transport, and slaughterhouse, as well as neck skin samples. This comprehensive sampling approach provides an integrated view of *Campylobacter* presence in intensively reared broiler chickens, from chick arrival to the final chilled product. It also enables comparison with the process hygiene criterion currently in force within the EU.

On farms, *Campylobacter* positivity rate in pooled cloacal samples was 9.4%, with detection emerging from the second week of age and peaking between days 15 and 22 (T2 and T3, both at 2.6%), followed by a decline to 1.5% at T4, with all samples testing negative at slaughter age (T5). Litter material assessed using disposable shoe-cover swabs, showed a substantially higher overall positivity rate of 23.5%, peaking at T3 (8.8%), with sporadic detection at the remaining sampling time points (2.9%). These findings are consistent with the colonization pattern described in the literature [20,29], where initially negative flocks rapidly become colonized from the second week onward, with efficient dissemination occurring via direct contact and the house environment. This is consistent with the litter positivity rate pattern observed in this study, as disposable shoe cover swabs directly sample the litter environment where cecal and cloacal excreta accumulate, making them highly sensitive to changes in shedding intensity and therefore closely mirroring the peak of flock colonization, a temporal association was supported by a moderate-to-strong positive Pearson correlation between cloacal and litter positivity rates (r = 0.652), although the limited number of paired time points (n = 6) constrains the statistical power of this analysis. Taken together, these findings suggest that litter material may act as a reservoir for *Campylobacter* on farm, in contrast to drinking water under the conditions assessed in this study.

A limitation of this study relates to the environmental sampling methodology. Although the disposable shoe cover was worn throughout each visit and a zig-zag walking pattern was applied to ensure broad contact with the litter, this approach may limit comparability with standardized boot swab protocols used for *Salmonella*. In addition, swabbing the shoe cover instead of directly homogenizing it in buffered peptone water may reduce detection sensitivity. While sterile swabs with Amies charcoal medium were used to preserve *Campylobacter* viability during transport, some variability in bacterial recovery may still occur. These factors should be considered when interpreting the results.

The higher overall positivity rate observed in litter compared to pooled cloacal samples is consistent with reports from European broiler production systems in which environmental matrices, including litter and boot sock samples, frequently present *Campylobacter* detection rates comparable to or exceeding those of pooled cloacal swabs, particularly from the third week of the production cycle onward [30]. This pattern likely reflects the integrative nature of environmental sampling methods, which capture diffuse contamination accumulated across the house floor rather than from individual animals [31]. The fact that all drinking water samples were negative suggests that, under the specific conditions of farms A, B, and C, the water system was not a critical point of *Campylobacter* introduction. This contrasts with findings from other studies that identify water as a relevant source under conditions of suboptimal water treatment or persistent biofilms in distribution lines [32,33]. These results indicate that biosecurity interventions should be more strongly directed toward controlling the movement of people and equipment, improving litter management, and reinforcing vector control measures, rather than prioritizing immediate changes to the water system. Nevertheless, periodic monitoring of litter should be maintained [31].

During transport, detection of *Campylobacter* in crate samples (15.0%), including positive identification both before and after transport, demonstrates that crates can act as a reservoir and major source of cross-contamination between batches when not adequately sanitized. However, the absence of *Campylobacter* in all crate samples analyzed after washing and disinfection in the second production cycle indicates that the applied cleaning and disinfection protocol effectively eliminated detectable contamination. These findings align with European recommendations that identify transport as a key factor in reducing infection pressure at slaughterhouse entry [2,34]. From a sector competitiveness perspective, the standardization of crate sanitization procedures, along with proper documentation and verification, can be effectively integrated into companies’ self-control programs.

At the slaughterhouse, the results reveal a paradoxically “good, yet improvable” situation. Only 8.3% of water samples were positive for *Campylobacter*, all collected after slaughter, suggesting that stunning, scalding, and washing systems did not function as primary sources of contamination, but rather as sporadic points of receipt of the agent released by carcasses. These findings suggest that, under evaluated conditions process water contamination was limited and associated with contact with contaminated carcasses during slaughter. This reinforces the importance of appropriate slaughter sequencing and evisceration control to prevent dissemination, as well as careful management of water temperature and carcass exposure time. This is consistent with evidence indicating that the main cross-contamination route in slaughterhouses is associated with processing stages such as evisceration and carcass-to-carcass contact, rather than with pathogen multiplication in process water, provided that temperature control and water renewal parameters are adequately maintained [2,29].

Notably, no published study has simultaneously characterized *Campylobacter* contamination across stunning, scalding, and washing water within the same facility and flock cohort, limiting direct comparisons. When examined independently, available data support this interpretation: scalding water counts of 4.22 ± 0.56 log CFU/mL at mid-day and 3.92 ± 0.59 log CFU/mL at end-of-day were reported, both undetectable at shift start following cleaning and disinfection, while washing water remained consistently negative across all sampling periods [35]. Under less-controlled conditions, positivity rates of 10.0% in scalding water and 27.5% in carcass rinse water were attributed to progressive carcass-to-water transfer during evisceration rather than to autonomous pathogen amplification [36]. Situated within the longitudinal follow-up conducted in the present study from day 0 through to slaughter, these findings consistently support the interpretation that process water functions as a passive recipient of carcass-derived microbial load, whose amplifying role is effectively curtailed when operational parameters are adequately maintained.

On the other hand, 76.7% of pooled neck skin samples were positive for *Campylobacter*, although most counts remained below 1000 CFU/g. From a legal standpoint, these results indicate compliance with the process hygiene criterion, in contrast to 15.2% of positive pools that exceeded the 1000 CFU/g threshold. Consequently, the number of “unsatisfactory” results would be 0 out of 50 in each theoretical sampling plan, which is below the maximum of 10 out of 50 permitted from 2025 (Regulation EU 2017/1495). However, from a public health perspective, the combination of high prevalence of positive samples with moderate contamination levels still represents a relevant source of consumer exposure. According to EFSA models, this interpretation shows that further reductions in average *Campylobacter* loads, even when already below 1000 CFU/g, can lead to a meaningful decrease in the incidence of campylobacteriosis [2]. Comparison between cycles also revealed that farms A and C showed increased skin loads in production cycle 2, with several pooled samples reaching 200 to 2600 CFU/g. In contrast, farm B showed a reduction in both the number of positive samples and bacterial counts during the same period. This contrast may reflect seasonal variation, as production cycle 2 took place during July and August, as well as differences in farm management and biosecurity practices. This pattern is consistent with the recognized seasonal epidemiology of *Campylobacter* in broiler production, where increased prevalence during the summer months is considered a well-established risk factor [2]. The elevated loads observed in farms A and C during cycle 2 (July–August) are therefore consistent with this expected summer peak, whereas the contrasting trend in farm B suggests that farm-specific management and biosecurity measures can outweigh the seasonal effect [31].

Notably, the marked increase in *Campylobacter* positivity rate between the farm stage, where pooled cloacal swabs showed an overall prevalence of only 9.4% and were entirely negative by the last on-farm sampling point (T5), and the slaughterhouse stage, where 76.7% of neck skin samples tested positive, warrants further consideration. This discrepancy is unlikely to reflect a true absence of colonization at flock level since *Campylobacter* shedding is known to be intermittent and pooled cloacal sampling has comparatively low sensitivity for detecting low-level or transient colonization [6]. Feed withdrawal, crafting and transport-associated stress immediately before slaughter are also recognized triggers of increased fecal shedding that would not have been captured by on-farm sampling performed earlier in the production cycle. Cross-contamination during slaughter operations is a further major contributor to carcass contamination, with processing steps such as scalding, defeathering, and evisceration substantially increasing *Campylobacter* loads independently of the initial colonization status of the flock [10]. This is consistent with previous findings linking carcass contamination not only to flock prevalence but also to processing-related factors [7]. These findings reinforce that on-farm prevalence alone is a poor predictor of final carcass contamination, underscoring the importance of process hygiene control at the slaughterhouse independent of upstream flock status.

From the slaughterhouse perspective, these data support the importance of considering the animal origin, rather than solely on intrinsic processing parameters. This approach is consistent with European recommendations on pre-slaughter interventions and the segmentation of processing lines according to flock *Campylobacter* status [2,7].

The predominance of *Campylobacter jejuni* (46.4%), followed by *C. coli* (32.1%) was consistent with the European context, in which *C. jejuni* is the species most frequently associated with broiler chickens and human infections [20,29]. Distribution of isolate origins, namely from neck skin, cloacal disposable shoe cover swabs, crates, and a small fraction from scalding/stunning water, further highlights the role of chilled carcass as the main point of risk concentration from a consumer perspective. This observation supports selecting neck skin as the regulatory sampling matrix.

Considering that poultry production is widely recognized as a significant reservoir of antimicrobial-resistant bacteria, including *Campylobacter*, it represents an increasing threat to global public health. The detection of *tet*O and *bla*_OXA-61_ in *C. jejuni* and *C. coli*, confirms that a considerable proportion of the *Campylobacter* population circulating on these farms and in the slaughterhouse carries resistance determinants relevant to human and veterinary therapeutics [13,37]. In particular, the recorded use of doxycycline in farm B during cycle 1, associated with the presence of *tet*O in isolates from the same farm, illustrates the direct link between antibiotic pressure and selection of resistant strains, consistent with international studies relating tetracycline use in poultry production with high prevalences of tetracycline resistance (88.9% in human and 72.4% in poultry isolates), the presence of the *tet*O gene (80.9%), beta-lactamase genes (specifically *bla*_OXA_ genes detected in 46.8% of isolates), and multidrug resistance (88.9% in human-derived and 82.8% in poultry-derived isolates) in both animal and human isolates [28]. This highlights the need to integrate *Campylobacter* control strategies with antimicrobial reduction and prudent use policies, as outlined in frameworks, such as the European One Health strategy.

## 5. Conclusions

This study addressed a critical knowledge gap regarding the dynamics of campylobacteriosis in intensive production systems, including the entire chain from farm to slaughterhouse. These findings show that *Campylobacter* colonization begins in the second week of broiler production, with litter and cloacal samples identified as the main on-farm reservoirs. Transport can contribute to dissemination, but the risk can be reduced through effective washing procedures, proper crate storage, and adequate vehicle disinfection, all of which help mitigate the presence of microorganisms. Slaughterhouse neck skin samples met the 1000 CFU/g criterion, despite a high percentage of positive-*Campylobacter* samples. s Additionally, a significant proportion of isolates carried resistance genes to tetracyclines and β-lactams.

Overall, this study reinforces the importance of implementing integrated control strategies that combine improved farm biosecurity, rigorous crate sanitization, optimization of evisceration and scalding procedures, systematic monitoring of *Campylobacter* in neck skin samples, and antimicrobial stewardship programs, thereby contributing to food safety and public health.

## Figures and Tables

**Table 1:** Nucleotide sequences with annealing temperature (AT) and expected amplicon size, used in this study for *Campylobacter* identification.

Primers	Nucleotide Sequence (5′ to 3′)	Size (bp)	AT (°C)	Reference
CH412F C1228R	GGATGACACTTTTCGGAGC CATTGTAGCACGTGTGTC	816	58	[24]
CC18F CC519R	GGTATGATTTCTACAAAGCGAG ATAAAAGACTATCGT CGCGTG	502	60	[25]
C1 C3	CAAATAAAGTTAGAGGTAGAATGT CCATAAGCACTAGCTAGCTGAT	161	58	[23]

**Table 2:** Nucleotide sequences with annealing temperature (AT) and expected amplicon size, used in this study for antimicrobial resistance gene detection.

Primers	Nucleotide Sequence (5′ to 3′)	Size (bp)	AT (°C)	Reference
tetO	GCGTTTTGTTTATGTGCG ATGGACAACCCGACAGAAG	559	56	[26]
blaOXA-61	AGAGTATAATACAAGCG TAGTGAGTTGTCAAGCC	372	54	[27]

**Table 3:** Number of samples collected by each farm-to-slaughter chain analyzed in this study.

Farm	Production Cycle *	Farm			Crate Transporte			Slaughterhouse				Total
		Cloacal	Litter	Drinking Water	Before	After	After Washing	Stunning Water	Scalding Water	Carcass Washing	Neck Skins	
**A**	**1**	300	6	6	4	4	0	2	2	2	50	376
	**2**	300	6	6	4	4	4	2	2	2	50	380
**B**	**1**	250	5	5	4	4	0	2	2	2	50	324
	**2**	300	6	6	4	4	4	2	2	2	50	380
**C**	**1**	250	5	5	4	4	0	2	2	2	50	324
	**2**	300	6	6	4	4	4	2	2	2	50	380
**Total**		1700	34	34	24	24	12	12	12	12	300	2164

* Cycle 1: November–December 2024, Cycle 2: July–August 2025.

**Table 4:** Total *Campylobacter* level obtained from pooled neck skin samples analyzed in this study, expressed as colony-forming units per gram (CFU/g) of neck skin, by production cycle 1 (November–December 2024) and cycle 2 (July–August 2025), for each farm. SD: standard deviation.

Pooled Neck Skins	Farm A		Farm B		Farm C	
	Cycle 1	Cycle 2	Cycle 1	Cycle 2	Cycle 1	Cycle 2
1	0	200	300	0	0	200
2	100	100	200	100	500	1200
3	1000	200	200	0	200	2600
4	0	400	100	0	0	2000
5	700	100	0	0	200	700
6	0	2100	100	200	100	200
7	100	200	100	200	200	800
8	100	600	200	0	100	2000
9	200	400	300	300	0	400
10	1000	400	200	0	0	800
Mean value/Cycle ± SD (CFU/g)	320 ± 413	470 ± 595	170 ± 95	80 ± 114	130 ± 157	1090 ± 839
Mean value/Farm ± SD (CFU/g)	395 ± 505		125 ± 112		610 ± 766	

## Data Availability

The original contributions presented in this study are included in the article. Further inquiries can be directed to the corresponding author.

## Abbreviations

The following abbreviations are used in this manuscript:

EFSA	European Food Safety Authority
ECDC	European Centre for Disease Prevention and Control
EU	European Union
CFU	Colony-forming units
PCR	Polymerase chain reaction
ISO	International Organization for Standardization
IRB	Institutional Review Board
BB	Bolton broth
BPW	Buffered peptone water
mCCDA	Modified Charcoal Cefoperazone Deoxycholate Agar
AT	Annealing temperature
bp	Base pairs
DNA	Deoxyribonucleic acid
